# Noninvasive estimation of oxygenation index in pediatric critical care: an independent retrospective observational validation

**DOI:** 10.3389/fped.2025.1675130

**Published:** 2025-10-02

**Authors:** Thomas E. Bachman, Christopher J. L. Newth, Nimesh Patel, Patrick A. Ross

**Affiliations:** ^1^ Department Biomedical Technology, Faculty of Biomedical Engineering, Czech Technical University in Prague, Kladno, Czechia; ^2^ Department Anesthesiology Critical Care Medicine, Children’s Hospital Los Angeles, University of Southern California Keck School of Medicine, Los Angeles, CA, United States; ^3^ Department of Anesthesiology, Vanderbilt University Medical Center, Nashville TN, United States

**Keywords:** hypoxemia severity, oxygen saturation, oxygenation index, pediatric critical care, validation & verification (V&V) 40

## Abstract

**Objective:**

To independently validate an empirically optimized algorithm for calculating estimated Oxygenation Index (eOI) using noninvasive parameters from pediatric intensive care populations.

**Design:**

Retrospective observational cohort study using an integrated patient data repository spanning over 12 years (August 2012-December 2024).

**Setting:**

Single tertiary children's hospital with general pediatric ICU (PICU) and cardiothoracic ICU (CTICU).

**Key measures:**

Arterial blood gas measurements were paired with coincident SpO_2_, heart rate, pulse rate, FiO_2_, and mean airway pressure measurements. The primary analyses used SpO_2_ observations between 80%–100%. Using these values eOI was calculated. The primary outcome was the Bias and Limits of Agreement of the difference between measured OI and eOI. Discrimination performance of eOI for severity of hypoxemia was evaluated using receiver operating characteristic curves at OI thresholds of 4, 8, and 16.

**Results:**

Analysis included 68,915 observations from 7,109 subjects (44,133 CTICU, 24,782 PICU observations). Bias was minimal in both populations: PICU 0.06 (95% CI; 0.03, 0.10) and CTICU 0.12 (95% CI; 0.09, 0.14). Limits of agreement were −5.2 to 5.4 (PICU) and −4.9 to 5.2 (CTICU). Discrimination performance was excellent, at 3 hypoxemia thresholds (AUROC; 0.91–0.98), and in the CTICU for OI ≥4 when SpO_2_ >97% (AUROC; 0.83).

**Conclusions:**

The new eOI algorithm provides accurate, but not precise, estimation of OI in both general pediatric and cardiothoracic ICU populations. Noninvasive OI monitoring may be shown clinically useful.

## Background

Optimizing the oxygenation of children in critical care is a fundamental task. Oxygenation is managed by careful titration of inspired oxygen. For those receiving mechanical ventilation establishing and maintaining appropriate lung recruitment requires titration of airway pressure, which is also essential. The adequacy of oxygenation is verified periodically with the measurement of oxygen tension and oxygen saturation in an arterial blood sample (PaO_2_, SaO_2_). Importantly, the saturation of oxygen is also noninvasively monitored continuously with a pulse oximeter (SpO_2_). While SpO_2_ is only an estimate of SaO_2_ and a reflection of the adequacy of PaO_2_, most titrations of the mean airway pressure and inspired oxygen are made based on trends in the SpO_2_.

Exposure to high and low PaO_2_ are associated with mortality and morbidity, as are inappropriate levels of mean airway pressure (mPaw) and inspired oxygen (FiO_2_). Oxygenation Index (OI) is a metric that integrated these parameters [(FiO_2_*100/PaO_2_)*mPaw]. It has become the accepted measure of severity of hypoxemia (1). When PaO_2_ values are not available, Oxygen Saturation Index (OSI: [(100*(FiO_2_/SpO_2_)*mPaw]), has been shown to be a suitable alternative (2). However, OSI has limitations. Primarily it is not reliable when SpO_2_ is >97%. Furthermore, while highly correlated to OI, OSI values are lower than OI values and thus the thresholds for severity are different. These indexes are currently used for a variety of purposes. These include predicting mortality, categorizing severity of disease, following the impact of therapeutic intervention, and setting thresholds for more invasive or aggressive therapies (1–4).

The relationship between SaO_2_ and PaO_2_ is not linear. Rather the relationship, referred to as the oxyhemoglobin dissociation curve, is sigmoid. This nonlinearity results in the insensitivity of high SaO_2_ levels to changes in PaO_2_. At any given SaO_2_, the corresponding PaO_2_ also shifts based on fluctuating physiological parameters such as pH and PaCO_2_. The SaO_2_-PaO_2_ relationship was quantified more than 4 decades ago by Severinghaus (5). More recently Gadrey and colleagues modified the Severinghaus equation for estimation of PaO_2_ using SpO_2_ rather than SaO_2_ (4). Based on a population of nearly 500 children receiving noninvasive respiratory support, they reported that the imputed PaO_2_ was effective in assessing the severity of hypoxemia for use in an organ failure metric.

Subsequently in 2021, Sauthier and colleagues reported on the outcome of an extensive project to determine the best approach to estimate PaO_2_ based on SpO_2_ (6). They used 4 machine learning approaches; three neural networks and an empirical bootstrap optimization of the Gadrey equation. This project was based on over 50,000 observations from a single PICU. The empirical approach not only refined the constants in the Gadrey equation but also excluded unreliable SpO_2_ measurements. The reliability exclusions were based on an observed difference between the pulse rate and the electrical heart rate. The performance of these 4 approaches, plus three previously described equations were then compared in an independent sample of over 12,000 observations from a different PICU. They concluded that their bootstrap equation was the most effective. Additionally, they reported that using the estimate of PaO_2_ to calculate estimated OI (eOI) was suitable for use with SpO_2_ values >97%, and also corresponded directly with OI values. They further suggested that availability of a continuous estimated OI would be a valuable addition to decision support systems and bedside management.

Critical care at Children's Hospital of Los Angeles is supported with a robust patient data management repository and has been active in developing and implementing decision support systems (7–12). This study aimed to confirm the performance of the Sauthier algorithm for calculating eOI in our general pediatric ICU and also to determine if it was effective in the cardiothoracic ICU.

## Methods

### Design

This is a retrospective observational cohort study of an existing integrated patient and treatment information data repository at a single tertiary level children's hospital. All data is automatically retrieved and archived. Use of masked aggregated information from this medical records database was approved for this project by the institutional review board (approved 2/21/22 by CHLA-IRB, CHLA-23-0019, Implications of Hyperoxemia and Hypoxemia in ICU Patients). The study was conducted in compliance with the ethical standards of the IRB and the Helsinki Declaration of 1975.

### Population

The database accessed reflects care in the 24-bed cardiothoracic unit (CTICU) and the 30-bed general pediatric unit (PICU). At the time of the query the entire database spanned a period starting in August 2012 and ending in December 2024. Admissions with at least one arterial blood gas assessment with a corresponding SpO_2_, FiO_2_ and mPaw measurement were identified and included in the analysis. In our ICUs this implies invasive conventional mechanical ventilation.

### Analysis parameters

Arterial oxygen tension (PaO_2_) measurements were paired with 5 other coincident parameters. These were peripheral oxygen saturation measurements (SpO_2_), heart rate, pulse rate, inspired oxygen (FiO_2_) and mean airway pressure (mPaw). The value used for each of these 5 parameters was the mean of 30-s values within ±1 min of the arterial sample point of care analysis. Observations with SpO_2_ < 80% were excluded, to be comparable to the development and validation set used by Sauthier et al. (6) However, after review of the results, a *post hoc* exploratory analysis of SpO_2_ observations in the CTICU with SpO_2_ between 70%–79% was conducted.

### Endpoints

The estimated PaO_2_ (ePaO_2_) was calculated according to the Sauthier algorithm (6). This calculation requires exclusion of observations with a difference in heart and pulse rate of 3% or more. The exclusion is based on the premise that when the measured pulse rate varies from the electrical EKG signal, the peripheral pulse, and thus the derived SpO_2_, is unreliable. The 3% threshold was derived by them empirically. We then calculated the estimated OI (eOI) using the ePaO_2_.

The primary outcome measure was the difference between the OI and eOI.

### Statistical analysis

The PICU and CTICU observations were treated as separate populations and all analyses were conducted in each separately.

We utilized the Bland-Altman paradigm for comparing methods for our analyses (13). Thus, the differences between the actual and estimate endpoint (OI) was characterized by two parameters. These were the Bias (mean difference) and the Limits of Agreement (95% confidence interval of the differences). The former reflecting accuracy and the latter precision. In addition, the 95% standard error of the mean of the Bias was also calculated. Finally, to evaluate these parameters across the range of oxygen saturations, the Bias and Limits of Agreement were calculated for 4 SpO_2_ strata (80%–87%, 88%–93%, 94%–97%, 98%–100%). For the *post hoc* analysis they were calculated for SpO_2_ between 70%–79% in the CTICU.

Receiver operator curves (ROC) were constructed to evaluate the predictive ability of eOI at three thresholds of hypoxemia. The area under the curves was calculated as the metric of effectiveness (AUROC). These thresholds were mild hypoxemia (OI, ≥4), moderate hypoxemia (OI, ≥8) and severe hypoxemia (OI, ≥16) (1). The first and last were used in Sauthier's validation (6). The moderate threshold was recently identified as prognostic (12).

Continuous descriptive data is presented as median and IQR (25th–75th percentiles), and categorical data as count and percent. Univariate Mann–Whitney test and *Z*-test were used to compare the differences in ICU populations. A *p* < 0.05 was specified as statistically significant. The 95% confidence intervals of the mean of the Oxygenation Index Bias and AUROC were used to compare the differences among ICUs, SpO_2_ strata, with statistical difference implied when they did not overlap. Statistical tests were conducted with XLSTAT v11.5 (Lumivero, NY USA).

## Results

Over ninety thousand observations (90,596) were identified in the 12-year period ending December 2024. Of these, 21,681 (24%) were excluded because of excessive differences between the observed pulse rate and heart rate. The remaining 68,915 observations from children with SpO_2_ between 80%–100% were included in the primary analysis. These observations were associated with 3,801 subjects in the CTICU and 3,308 subjects in the PICU. Sixty-four percent of the observations (44,133) were from the CTICU.

An overview of the two ICU cohorts is shown in Table 1. Based on the threshold of OI <4, more than a third of the observations were not associated with significant hypoxemia, and nearly a half were between 4 and 16. Most of the parameters were statistically significantly different, however of note, those in the cardiothoracic unit were markedly younger with lower oxygen tensions, but less severe hypoxemia.

**Table 1:** Observations by ICU.

	CTICU	PICU	*p*
Subjects	3,801	3,308	na
Observations/subject	6 (3–14)	4 (2–4)	*P* < 0.001
Observations	44,133	24,782	na
Age (days)	2.0 (0.07–13)	105 (29–183)	*P* < 0.001
Weight (kg)	4.7 (3.3–8.8)	28 (13–50)	*P* < 0.001
PaCO_2_ (mmHg)	42 (38–47)	42 (36–50)	ns
pH	7.40 (7.36–7.44)	7.40 (7.34–7.44)	*P* < 0.001
FiO_2_ (%)	40 (35–61)	40 (30–60)	*P* < 0.001
PaO2 (mmHg)	81 (51–132)	90 (72–116)	*P* < 0.001
SpO_2_	97 (89–99)	98 (95–99)	*P* < 0.001
%>97 SpO_2_	48%	54%	*P* < 0.001
Paw (mean cm H_2_O)	10 (8.4–12)	13 (9.8–17)	*P* < 0.001
OSI^a^	4.7 (2.9–8.1)	8.0 (4.6–14)	*P* < 0.001
OI	4.7 (3.0–8.5)	5.4 (2.9–11)	*P* < 0.001
≥4	60%	62%	*P* < 0.001
≥16	9.4%	16%	*P* < 0.001

^a^ OSI not calculated when SpO_2_ > 97%.

Median and (IQR) for continuous and % for proportion.

**Figure 1:**
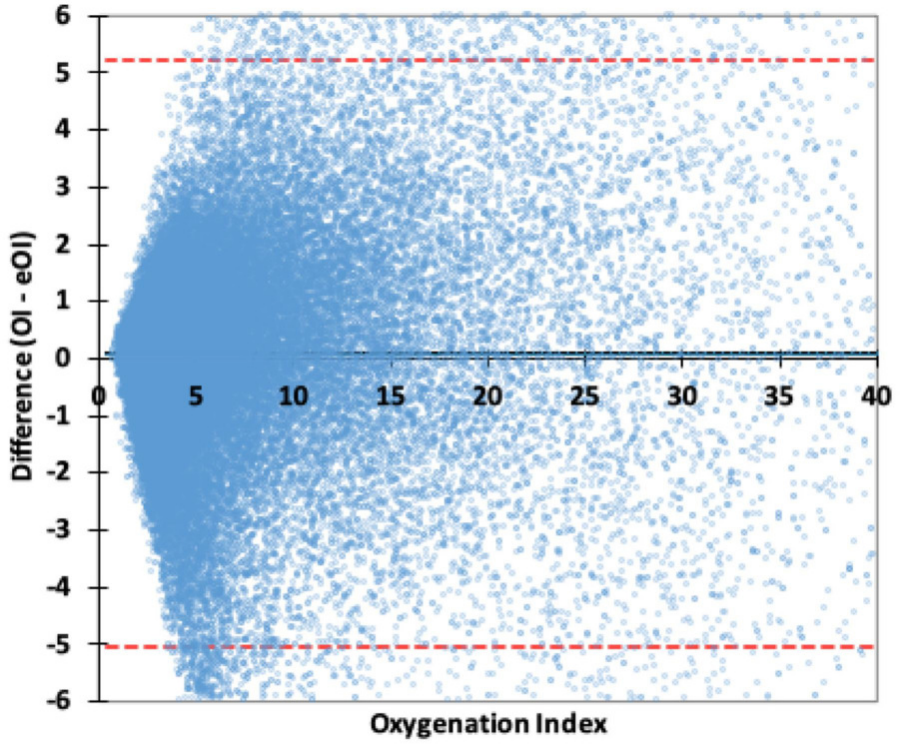
Bland–Altman plot OI difference. The red lines are the 95% Limits of Agreement, the wide black line the Bias.

Figure 1 is a Bland-Altman plot of the OI-eOI difference. While the Bias is very small (0.10), the Limits of Agreement are about 5. The primary analysis by ICU, found minimal Biases, that were not statistically different. The Bias in the PICU was 0.06 (CI: 0.03, 0.10) and in the CTICU it was 0.12 (CI: 0.09, 0.14). The Limits of Agreement were similar and predictably wide, −5.2 to 5.4 in the PICU and −4.9 to 5.2 in the CTICU.

These Bias and Limits of Agreement values for 80%–100% SpO_2_, as well as those for the 4 specified strata of SpO_2_ are detailed in Table 2. There were statistically significant differences in Bias among the strata and between the ICUs; however, the magnitudes of the Bias differences were all relatively small (<10% of the OI for that stratum). In the two strata of 93% SpO_2_ and below, the Bias was positive in the CTICU and negative in the PICU, while the inverse was true above 93%. Except for the 98%–100% strata, in the PICU the Bias was lower (OI was higher than the eOI). The Limits of Agreement were for most part similar between strata and ICUs. The *post hoc* analysis of 9,923 observations with SpO_2_ between 70%–79% in the CTICU found a Bias of 0.10 (CI: 0.02, 0.18), markedly smaller than the 80%–87% Bias but with slightly higher Limits of Agreement (−7.8 to 8.0).

**Table 2:** Bias and limits of agreement OI-eOI difference by SpO_2_ categories.

	CTICU	PICU
SpO_2_ 80%–100% (%)	44,142 (100%)	24,782 (100%)
OI (IQR)	4.7 (3.0–8.5)	5.4 (2.9–11)
Bias: OI-eOI (95%CL)	0.12 (CI 0.09, 0.14)	0.06 (CI 0.03, 0.10)
LOA OI-eOI (95%CL)	−4.9 to 5.2	−5.2, 5.4
SpO_2_ 80%–87% (%)	9,676 (22%)	572 (2%)
OI (IQR)	8.9 (5.3–16)	28 (20–38)
Bias: OI-eOI (95%CL)	0.76 (0.71, 0.80)	−2.6 (−3.1, −2.2)
LOA OI-eOI (95%CL)	−4.1, 5.6	−13, 8.3
SpO_2_ 88%–93%	4,761 (11%)	2,884 (12%)
OI (IQR)	7.3 (4.5–13)	18 (11–27)
Bias: OI-eOI (95%CL)	0.72 (0.65, 0.79)	−0.45 (−0.59, −0.31
LOA OI-eOI (95%CL)	−4.3, 5.7	−7.9, 7.0
SpO_2_ 94%–97%	8,552 (19%)	7,976 (32%)
OI (IQR)	5.2 (3.5–8.2)	7.7 (4.3–13)
Bias: OI-eOI (95%CL)	−0.12 (−0.18, −0.07)	0.13 (0.07, 0.18)
LOA OI-eOI (95%CL)	−5.2, 4.9	−4.8, 5.0
SpO_2_ 98%–100%	21,337 (48%)	13,360 (54%)
OI (IQR)	3.3 (2.3–5.1)	3.6 (2.3–6.1)
Bias: OI-eOI (95%CL)	−0.22 (−0.25, −0.18)	0.25 (0.21, 0.29)
LOA OI-eOI (95%CL)	−5.2, 4.8	−4.2, 4.7

Median (25–75th) and mean (2.5%, 97.5% SEM).

**Figure 2:**
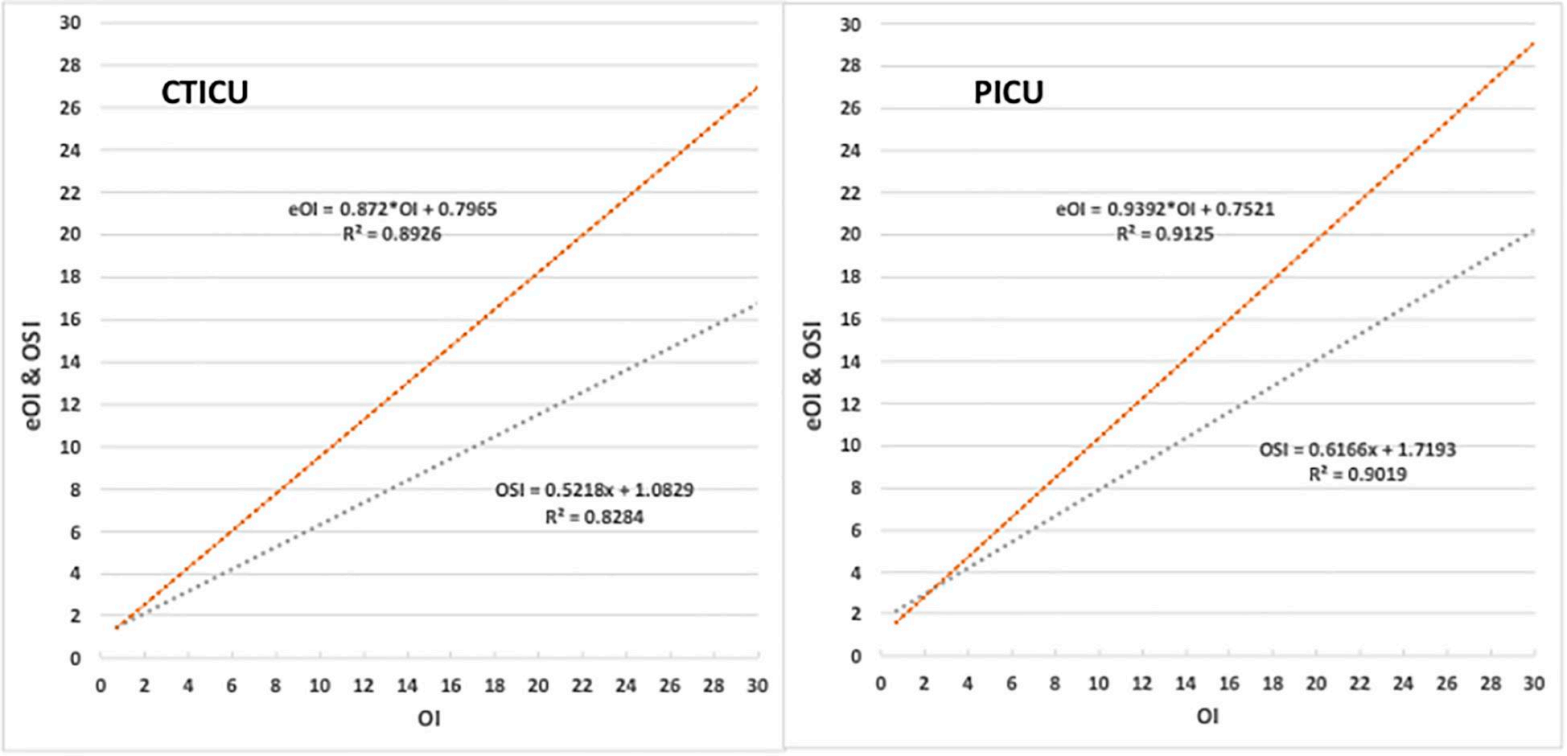
Comparison of eOI and OSI to OI. Panel on left is the CTICU, and the right PICU. Upper red line is eOI vs. OI, lower blue the OSI vs. OI.

Figure 2 provides a chart for each ICU comparing eOI and OSI to OI for SpO_2_ between 80%–97%. The coefficients of determination (R^2^) are high for eOI and OSI, in both ICUs. The coefficient of determinations in the PICU is marginally better than in the CTICU. In the CTICU the *R*^2^ for eOI (0.89) is also higher than for OSI (0.83), and comparable in the PICU. The eOI, unlike the OSI, clearly approximates the OI.

The ability of eOI to discriminate at thresholds of OI ≥4, OI ≥8 and OI ≥16 for both SpO_2_ above and below 97% SpO_2_ is excellent, as reported in Table 3. In the PICU the performance for both OIs is (AUROC between 0.933 and 0.985). In the CTICU the performance is similar (AUROC between 0.909 and 0.985) in all but OI ≥4 when SpO_2_ is >97% (0.830).

**Table 3:** Discrimination of hypoxemia severity using eOI (AUROC).

	CTICU	PICU
OI ≥4		
80%–97% SpO_2_	0.909 (0.904, 0.913)	0.973 (0.969, 0.977)
98%–100% SpO_2_	0.830 (0,825, 0.836)	0.933 (0.929, 0.937)
OI ≥8		
80%–97% SpO_2_	0.972 (0.970, 0.974)	0.978 (0.976, 0.980)
98%–100% SpO_2_	0.896 (0.890, 0.903)	0.952 (0.948, 0.980)
OI ≥16		
80%–97% SpO_2_	0.984 (0.982, 0.986)	0.985 (0.983, 0.987)
98%–100% SpO_2_	0.954 (0.943, 0.965)	0.965 (0.955, 0.974)

Area under the ROC mean (95% CI).

## Discussion

We evaluated the performance of a method of estimating OI using noninvasive parameters, in nearly 70,000 observations from our critical care populations. We verified its performance in our PICU, a population similar to the one from which it was developed and also subsequently validated. We also found it was effective in our CTICU, a new population in which it had not been tested.

Our results from our PICU were consistant with those of Sauthier's, though the populations were slightly different. The verification set used by Sauthier and colleagues was much smaller than our PICU verification set. (24,782 observations in 3,308 subjects compared to 12,047 observations in 926 subjects). Their validation population included a significant portion of patients receiving noninvasive respiratory support, whereas our population consisted entirely of mechanically ventilated patients. However, the median severity of hypoxemia of the two were similar (OI: 4.3 and 5.4). They reported a Bias in eOI of 0.13 above and 0.15 below a 97% SpO_2_. Overall, we found the bias to be slightly less than they reported. Between 80%−100% SpO_2_, the Bias in our PICU was 0.06 (CI: 0.03, 0.10). Our analysis by SpO_2_ strata, however, identified positive and negative biases among SpO_2_ strata, which were comparable to their results. The ability to discriminate standard levels of hypoxemia, was generally comparable based on the area under the ROC but slightly better in our data for an OI of 4 with a SpO_2_ of >97% (0.93 vs. 0.88).

Our report is to our knowledge the first evaluation of the equation in cardiothoracic subjects. Of note, while we did not exclude subjects with cyanotic heart anomalies, we only evaluated SpO_2_ observations of 70% or higher. In the aggregate, the Bias and Limits of Agreement were comparable to our findings in the PICU. There was a larger Bias when SpO_2_ was less than 93%. In the CTICU eOI tended to read lower than the actual OI, while it was slightly higher above 93%. This trend or slope was the opposite in the PICU. Variations among SpO_2_ strata were not available in the previous validation. Nevertheless, this small difference that we identified does not seem clinically relevant.

Our findings also support the conclusion of Sauthier and colleagues; that use of eOI offers advantages over OSI. Most importantly it may be useful in subjects with SpO_2_ levels greater than 97%, when OSI is not available. In addition, its values are consistent with OI, and also offers a marginally better coefficient of determination below 97%. This is not surprising in that the Sauthier algorithm not only excludes unreliable SpO_2_ readings, but also adjusts for the nonlinearity of the PO_2_-SO_2_ relationship. Of course, eOI requires more sophisticated calculations and cannot be *guesstimated* at bedside. Though, eOI and OSI provide excellent discrimination of hypoxemic severity, it should be reiterated that their lack of precision limits their use for therapeutic decisions. Another recent study of the use of this equation for calculating ePF ratios in children who were receiving noninvasive respiratory support also demonstrated similar advantages over SF ratios (14).

The advantage of integrating the SpO_2_, meanPaw, and FiO_2_ measurements and following an eOI on a monitor or decision support system is seductive. The question is whether it would have clinical utility. Certainly, the Bias of eOI is more than adequate for such a purpose. As noted, the lack of precision reflected in the Limits of Agreement is a significant concern for reliability. In a continuous derived signal, it would not be any more erratic than SpO_2_ can be at times, and could provide a useful trend in severity of hypoxemia. Likely it would be better with unreliable SpO_2_ readings not included in the calculations. Changes in pH, PCO_2_, and body temperature all shift the oxygen dissociation curve and practical matters associated with pulse oximeters accuracy (15) are also in play. Those are reflected equally in SpO_2_ and eOI values. We suggest that an integrated continuous eOI or ePF signal along with SpO_2_ might be an improvement but only if averaging minimized physiological noise reflected in the wide Limits of Agreement. Even so, the clinical utility of such a continuous monitored parameter requires further evaluation especially as it interacts with current PEEP guidelines recommended for pediatric ARDS.

Our study has a number of strengths. First the data in our database is reliable. It is automatically collected and not subject to transcription errors likely in older smaller studies. Nevertheless, the PaO_2_ measurements could not be precisely aligned with the SpO_2_ averages. Our verification of the eOI accuracy in the PICU is both independent from the developers and also a much larger sample population. Both supporting projectability to other centers. Our verification of its effectiveness in cardiothoracic subjects also extends its utility, potentially including SpO_2_ levels between 70%–80%. Our study also has some limitations. Our population were all receiving mechanical ventilation, although a good proportion did not meet the criteria for mild hypoxemia. The analytical approach we used did not account for multiple observations of subjects, however given the small proportion of repeated measurements and large sample size, we consider it unlikely to have had a substantial effect on the results. Finally, we only tested the discrimination effectiveness at OIs of 4, 8 and 16. Use as a monitor of severity of hypoxemia would require verification across a much wider range of OIs.

## Conclusion

We independently evaluated a new algorithm for estimating Oxygenation Index, that requires only noninvasive measurement readily available from today's patient data monitoring systems. We validated its performance in both critically ill general and cardiothoracic children. We found it suitable for assessing the severity of hypoxemia, and see potential for its use in continuous decision support systems. The latter requiring focused applied research.

## Data Availability

The raw data supporting the conclusions of this article will be made available by the authors, without undue reservation.
