# Supplementary material for: Noninvasive estimation of oxygenation index in pediatric critical care: an independent retrospective observational validation
Source: Front Pediatr. 2025 Oct 2;13:1675130. doi: 10.3389/fped.2025.1675130 (PMC12527840; doi:10.3389/fped.2025.1675130)
Supplement: Supplementary file 1 [file Datasheet1.pdf]

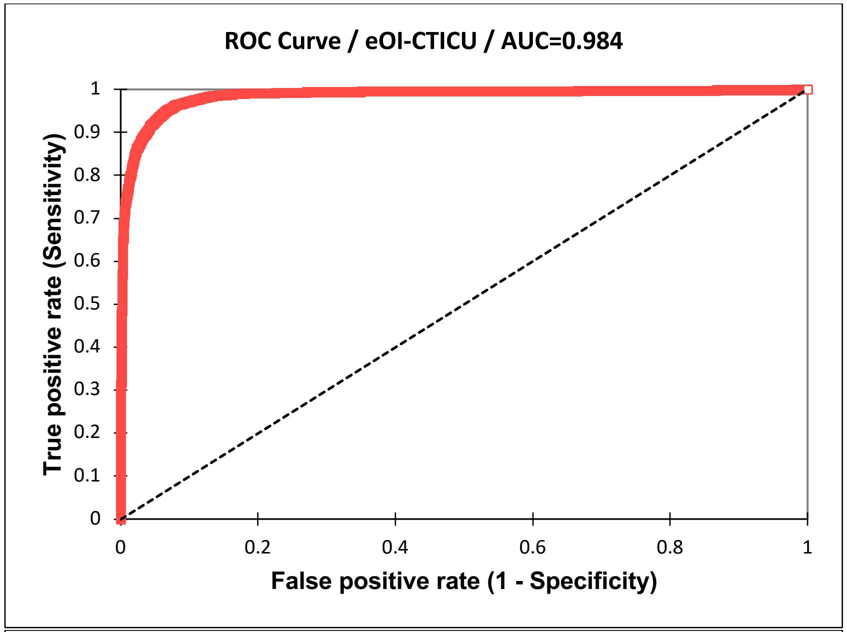
ROC Curve / eOI-CTICU / AUC=0.984
True positive rate (Sensitivity)
False positive rate (1 - Specificity)
0
0.1
0.2
0.3
0.4
0.5
0.6
0.7
0.8
0.9
1
0.2
0.4
0.6
0.8

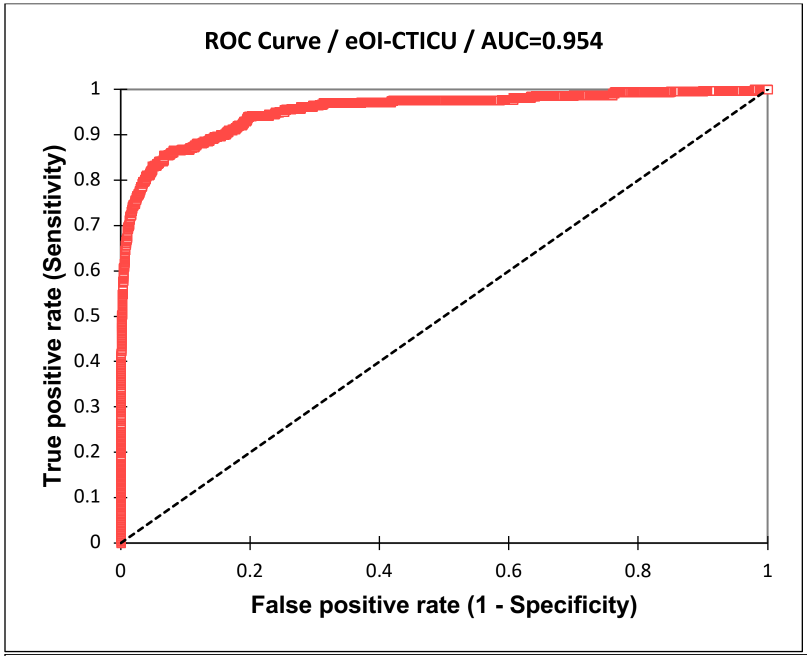
ROC Curve / eOI-CTICU / AUC=0.954
True positive rate (Sensitivity)
False positive rate (1 - Specificity)
0
0.1
0.2
0.3
0.4
0.5
0.6
0.7
0.8
0.9
1
0.2
0.4
0.6
0.8

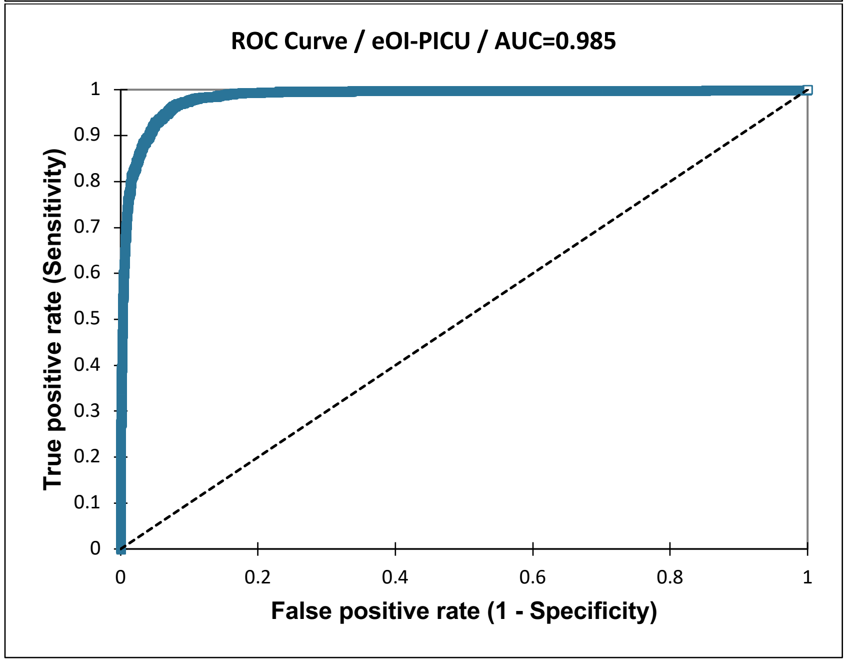
ROC Curve / eOI-PICU / AUC=0.985
True positive rate (Sensitivity)
False positive rate (1 - Specificity)
0
0.1
0.2
0.3
0.4
0.5
0.6
0.7
0.8
0.9
1
0.2
0.4
0.6
0.8

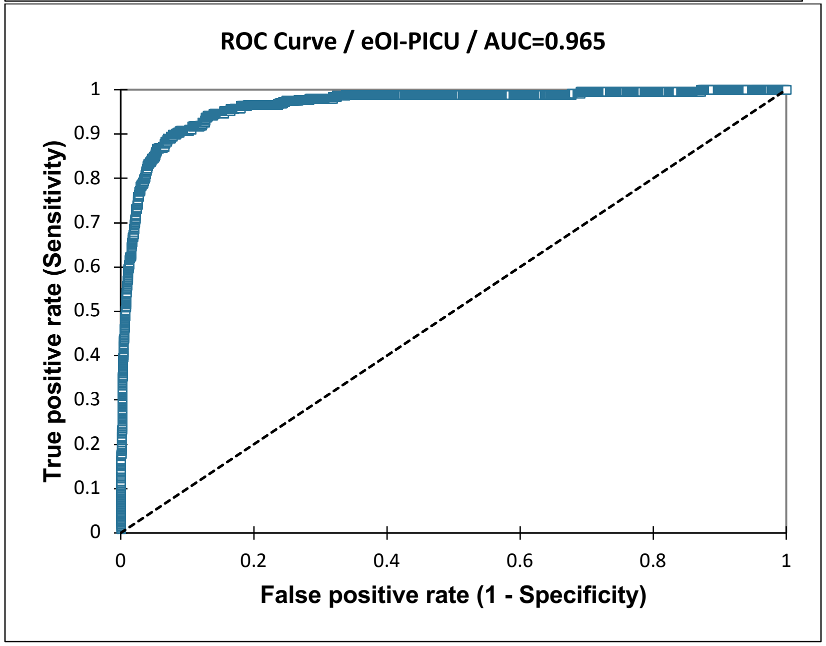
ROC Curve / eOI-PICU / AUC=0.965
True positive rate (Sensitivity)
False positive rate (1 - Specificity)
0
0.1
0.2
0.3
0.4
0.5
0.6
0.7
0.8
0.9
1
0.2
0.4
0.6
0.8

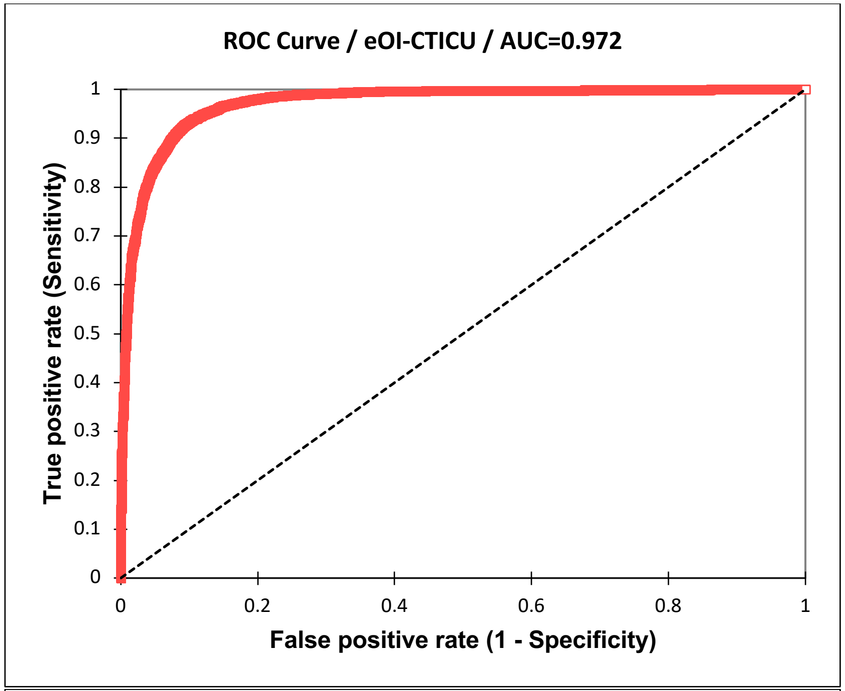
ROC Curve / eOI-CTICU / AUC=0.972
True positive rate (Sensitivity)
False positive rate (1 - Specificity)
0
0.1
0.2
0.3
0.4
0.5
0.6
0.7
0.8
0.9
1
0
0.2
0.4
0.6
0.8
1

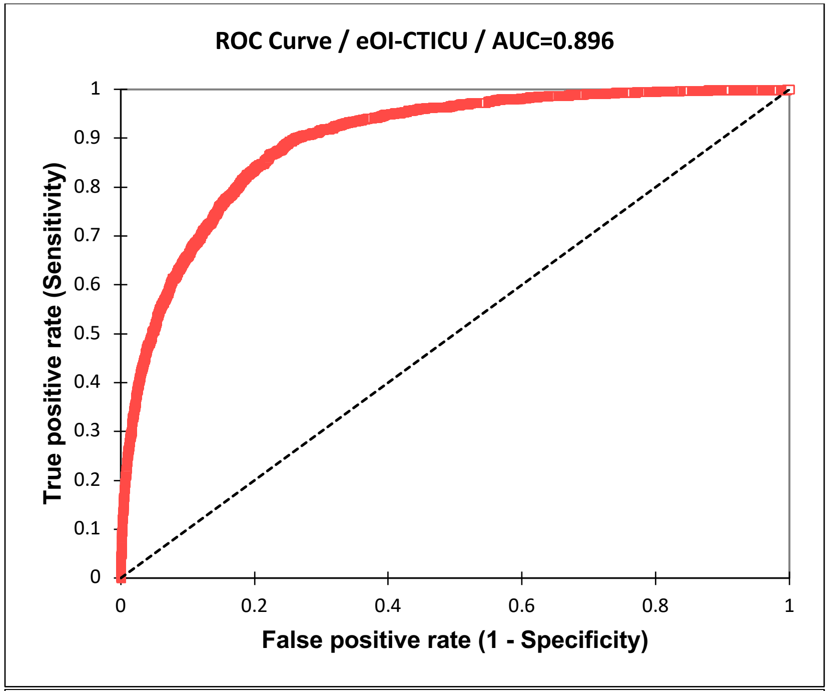
ROC Curve / eOI-CTICU / AUC=0.896
True positive rate (Sensitivity)
False positive rate (1 - Specificity)
0
0.1
0.2
0.3
0.4
0.5
0.6
0.7
0.8
0.9
1
0
0.2
0.4
0.6
0.8
1

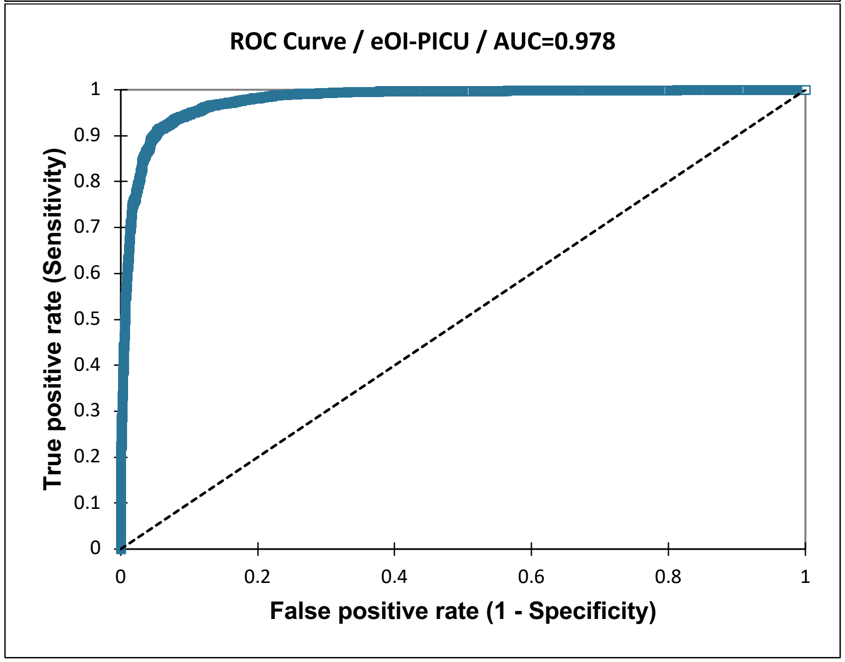
ROC Curve / eOI-PICU / AUC=0.978
True positive rate (Sensitivity)
False positive rate (1 - Specificity)
0
0.1
0.2
0.3
0.4
0.5
0.6
0.7
0.8
0.9
1
0
0.2
0.4
0.6
0.8
1

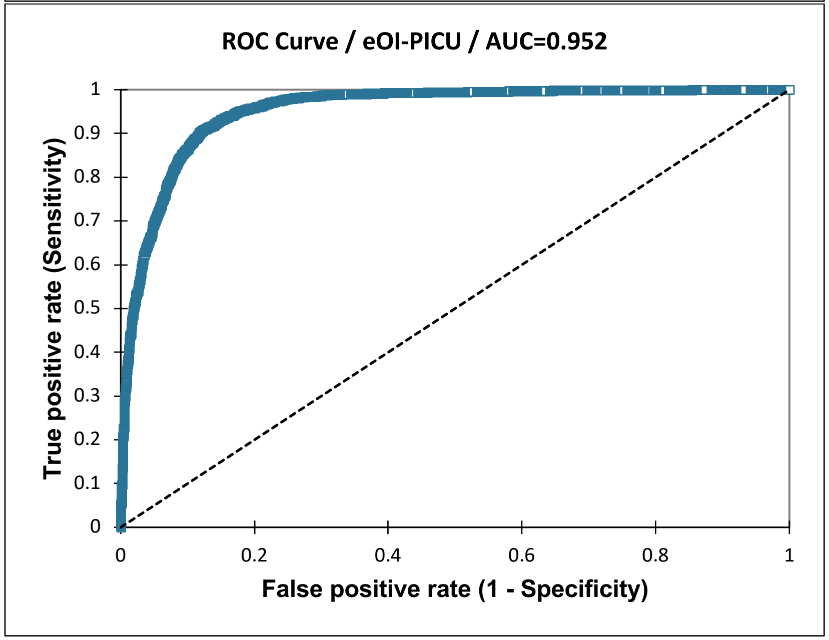
ROC Curve / eOI-PICU / AUC=0.952
True positive rate (Sensitivity)
False positive rate (1 - Specificity)
0
0.1
0.2
0.3
0.4
0.5
0.6
0.7
0.8
0.9
1
0
0.2
0.4
0.6
0.8
1

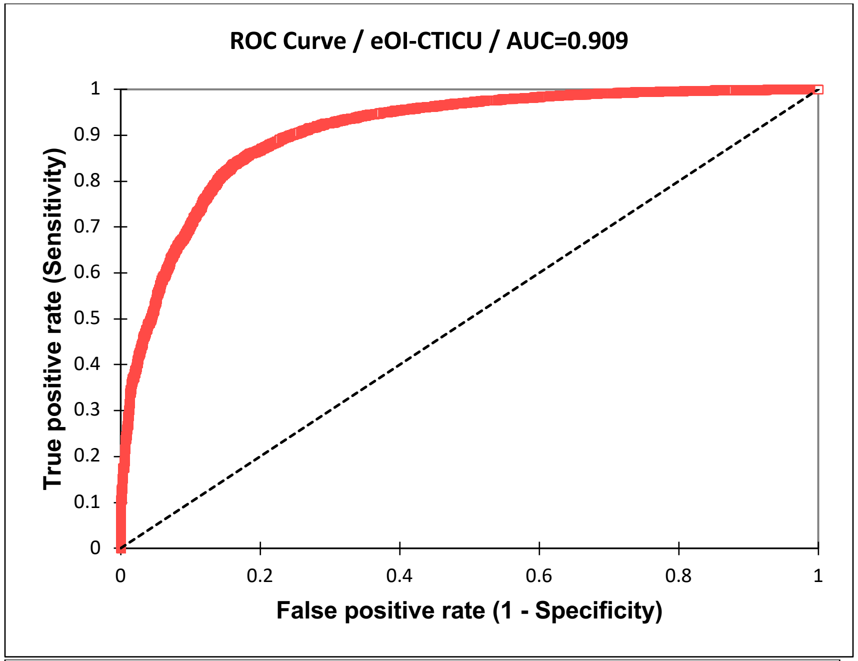
ROC Curve / eOI-CTICU / AUC=0.909
True positive rate (Sensitivity)
False positive rate (1 - Specificity)
0
0.1
0.2
0.3
0.4
0.5
0.6
0.7
0.8
0.9
1
0
0.2
0.4
0.6
0.8
1

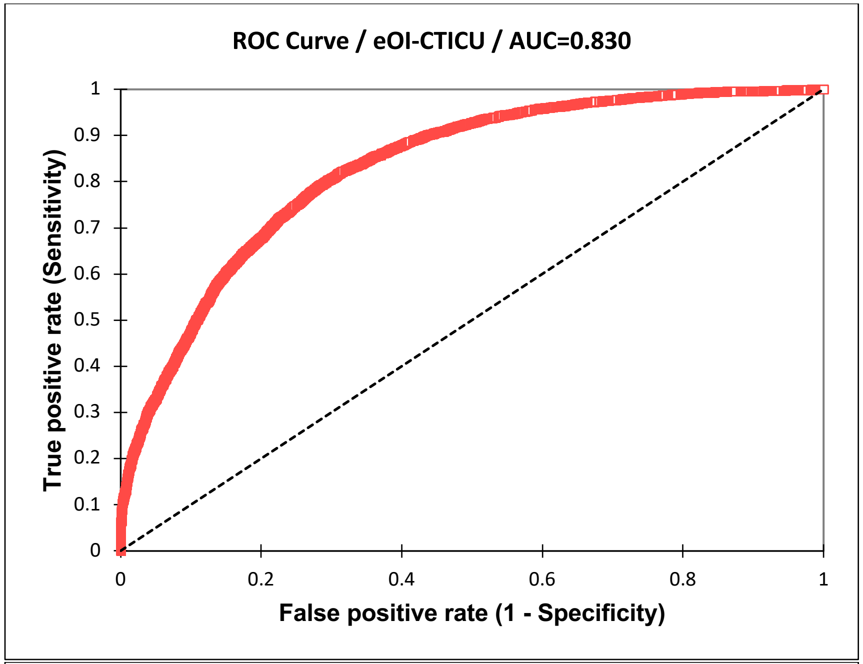
ROC Curve / eOI-CTICU / AUC=0.830
True positive rate (Sensitivity)
False positive rate (1 - Specificity)
0
0.1
0.2
0.3
0.4
0.5
0.6
0.7
0.8
0.9
1
0
0.2
0.4
0.6
0.8
1

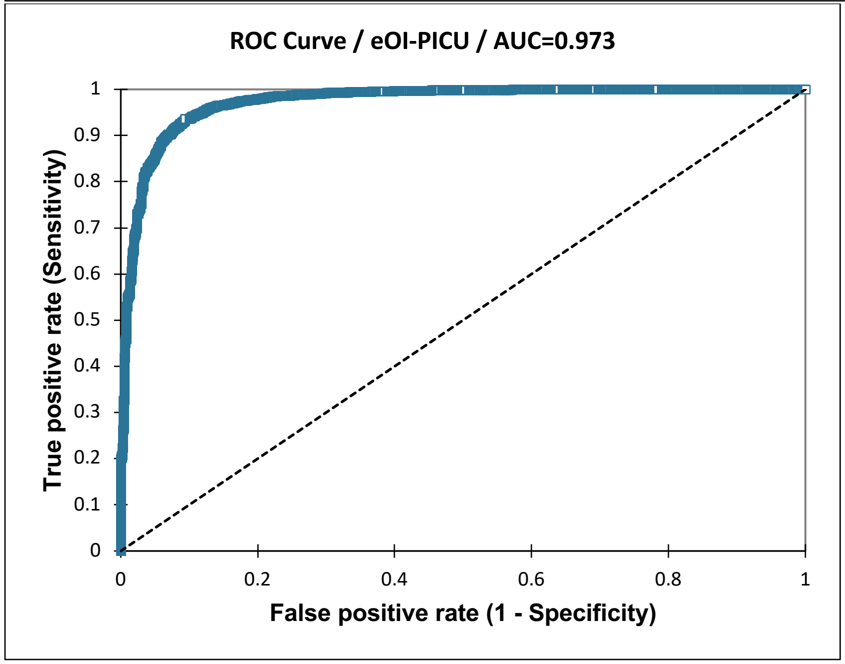
ROC Curve / eOI-PICU / AUC=0.973
True positive rate (Sensitivity)
False positive rate (1 - Specificity)
0
0.1
0.2
0.3
0.4
0.5
0.6
0.7
0.8
0.9
1
0
0.2
0.4
0.6
0.8
1

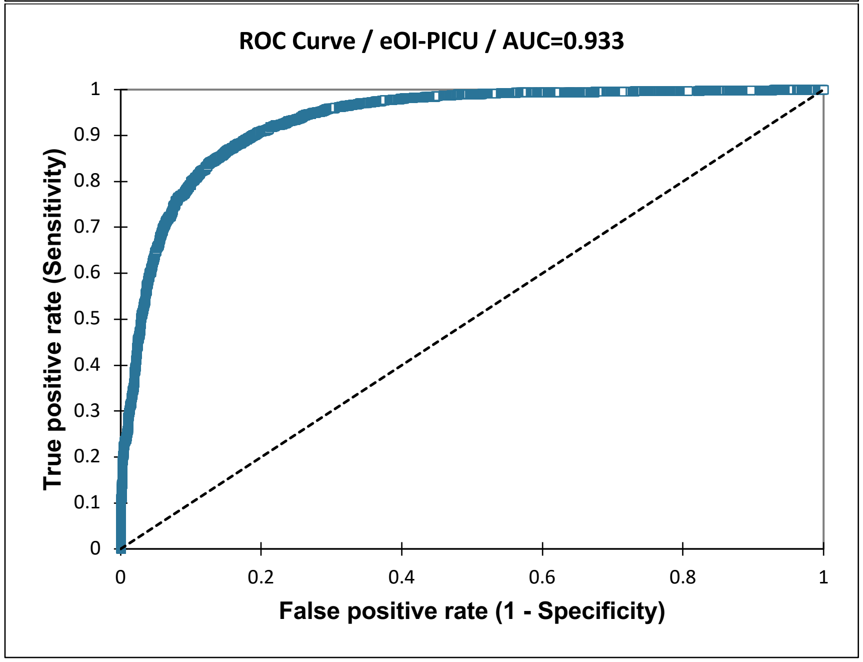
ROC Curve / eOI-PICU / AUC=0.933
True positive rate (Sensitivity)
False positive rate (1 - Specificity)
0
0.1
0.2
0.3
0.4
0.5
0.6
0.7
0.8
0.9
1
0
0.2
0.4
0.6
0.8
1

Supplementary Fig s1 Receiver Operator Curves

The top panel is OI≥16, middle panel OI≥8, and bottom OI≥4

The left column is for SpO2 80-97%, and the right SpO2 >97-100%

The red ROC curves are for the CTICU and the blue the PICU
